# Albany County Medical Society

**Published:** 1874-06

**Authors:** F. C. Curtis

**Affiliations:** Secretary


ART. III.—*Albany County Medical Society. Semi-Monthly Meeting held March 25th*, 1874. Reported by F. C. CURTIS, M. D., Sec'y.

The Society met at the City Building, on the 25th of March, at 8 P. M.

The President, Dr. SWINBURNE, in the chair.

Twenty-three members were present.

The name of Dr. C. S. MERRILL was proposed for membership, and referred to the *Comitia Minora*.

Dr. J. DE ZOUCHE read a paper on the subject of Hygiene in its relation to Hospitals.

"The philanthropic desire to establish a hospital does not always go hand in hand with the power to do so in any comprehensive manner, and the promoters are often compelled to begin in a small way, using a building intended for quite other purposes and requiring a great deal of expensive modification to make it half way suitable as a receptable for the sick. In one instance that I know of a new hospital was made of an old jail, and I need hardly say it needed all the genius of its promoters and plenty of money to make it at all suitable for the new kind of work to which it was devoted."

In illustrating the importance of thorough drainage and sewerage the doctor cited the fact that the German Hospital, cor., 77th street and 4th avenue, New York, was built on a site that included an old drainage stream (as we learn from the Report on Defective drainage by the committee of the State Med. Soc.) "Material poison undoubted from this source so universally affected the patients that it was in serious contemplation by its Trustees at one time to abandon its use for hospital purposes. So universal was the fact that patients entering it contracted malarial diseases that it became the ordinary practice to administer quinine to its inmates as a prophylactic immediately upon their admission!"

"Defective drainage," says the same report, "may be set down as the cause of one-fifth of the total mortality of Brooklyn."

The importance of proper ventilation, so necessary to the well, is vastly more important to the sick in hospitals. Air that has once been used in the act of breathing, bears the same relation to pure air that venous blood does to arterial. It has been contaminated by effete and poisonous elements, and must not be reinhaled.

"Now as at each act of ordinary breathing, about 25 cubic inches of air are changed in the lungs, and as this act is repeated about a thousand times in an hour, it follows that 25,000 cubic inches of pure air per hour are needed for healthy life. The inference is obvious. Provision must be made in hospital wards for the constant change of air, and estimates have been formed as to the cubic space which should be allotted to each patient, that sufficient pure air may be secured to them. Less than 1,200 cubic feet to each patient is deemed insufficient, and in one hospital named they allow 2,100 cubic feet and hardly deem that enough.

"In ordinary dwellings, where the inmates are as a rule healthy, the usual means of ventilation by windows and doors answer the purpose very well; but in a hospital, where medical and surgical diseases prevail, and the air beoomes laden with the foul exhalations from every variety of decaying tissue, it is of the utmost importance to take advantage of every means by which pure air may enter and the impure escape most effectively. Now, pure air, as a rule, enters a living-room at the lowest openings, whilst the air which has become vitiated by having passed through the lungs, or contaminated by contact with the exhalations from the surface of the diseased body, ascends to the top of the room, and will escape more or less completely by the highest openings. I qualify the statement, believing it will not wholly escape unless the highest openings are at the highest point in the room, on a level with the ceiling. The lower openings should be at or near the level of the floor. The exhaled air is largely composed of carbonic acid gas, which, at the same temperature as the pure air, is much heavier than it, volume for volume. When first exhaled, being expanded by heat, its specific gravity is less, and it ascends, to escape measurably by such openings as there may be; but if proper provisions have not been made above for its departure at this time, it quickly condenses and sinks to the lower part of the room, where it is destined to struggle with the pure air, provided there are inlets.

"Now, if there are openings at the level of the floor on all four sides of the room, there will be less opportunity for the accumulation of this poisonous gas, which would escape on the side of the room which sailors call "leeward," impelled by the pure air entering at "windward." The upper openings in the rooms should be somewhat larger than the lower, for obvious reasons; and I think it important that they should be at the level of the ceiling. When this is neglected, there will always be a reserve of deleterious gases found hovering about the angles, and tainting the whole upper stratum of air in the room, to the great detriment of the patients, to whom the oxygen of the pure air is even more essential than colomel or quinine."

The Pavillion style of building hospitals was discussed; the preference being given to it.

"So long as hospitals are located within city limits, where land is so valuable, there would seem to be no choice but to build several stories high, and to have overcrowded wards from time to time. Where such is the case the time invariably comes that the rate of mortality increases from causes growing out of the aggregation of sick and diseased. No amount of care has hitherto served to prevent the thorough infection of large hospital buildings, so far as I know—the rate of mortality slowly but steadily increasing in proportion to the degree of aggregation. Blood poisoning, in one or other of its various manifestations—hospital pyemia, erysipelas, &c., persistently claims constantly higher per centages of victims. You may whitewash the walls and ceilings a dozen times a year, and spend a fortune in disinfectants, but the fact will force itself on you that every year, notwithstanding the great advances made in surgery proper, the proportion of fatal cases becomes constantly larger."

"The remedy would seem to be to build your hospital where you can buy land by the acre and not by the foot. Take the plan of the Leipzig hospital for example, which is built, not in the heart of the city, but "at the gates." It consists of fourteen large "sheds"—if such a name can be given to what are really handsome pavillions. They are detached one-story frame buildings, connected through an ante-chamber by a gallery and facing a garden. They are 100 by 32 feet (inside), with a height of 15 feet to the eaves and 20 feet to the roof-ridge. They stand 60 feet apart, and are raised four feet high for the sake of ventilation. There are ventilators in the roof-ridge. There are isolated sheds for contagious diseases. It would be a wise bestowal of money if our commissioners of charities, and the various benevolent corporations that undertake from time to time the building of hospitals, were to put a larger share of their funds into more land, and a lesser share than they have hitherto done into architectural effects and elaborate costly ventilating fixtures that do not ventilate."

Dr. T. D. Crothers read a paper on Inebriation, its Pathology and treatment:

Modern investigations indicate that alcohol is not a stimulant in a literal sense, but a diminisher of vital force, suspending interstial activity, and resulting in vaso motor paralysis. This theory is now discussed and accepted by many of the leading pathologists. A brief review of its principles will not be out of place in this connection.

Alcohol affects the system in three separate physiological functions, viz: Circulation, Respiration, and Innervation.

On the circulation: Alcohol taken in the stomach causes increased influx of blood to the face, and integuments, and all the vascular surface supplied by the branches of the opthalmic artery. This is indicated by the flush and color of the face. This artery is the first branch of the carotid after its passage into the cranium, and indicates clearly the circulatory system within the brain. The heart's action is increased, and the arterial capilaries are dilated with the increased number of red corpuscles. This condition becomes intensified when continued doses of alcohol are given. The dilation goes on and the larger trunks of the veins are involved, and the heart's action diminishes in rapidity, but not in volumn.

On the respiratory system: Both inspiration and expiration are accellerated coincidently with the increased action of the heart. Although there are evidences of imperfect oxydations in the deepening color of the face, and turpidity of the venous system.

Effects of alcohol on innervation: The sense of burning in the stomach and face may be due to sensory innervation, or to change resulting from increased amount of oxygen carried by the red corpuscules to the terminations of the sensory nerves. Vaso motor activity is diminished, as seen in dilatation of the arteries. The functional power of the cerebral hemispheres is impaired as seen in the weakened will, and insubordination of the emotional centers and those of special sensation, causing impressions to be confused, inacurate and unreliable. Muscular motions are irregular, and incoordinate, and general innervation is reduced to its lowest expression. The functional energy of the superior ganglia of the nervous system is diminished gradually to nearly a negative existence.*

The physiological significance of these symptoms are apparent, when we consider the engorgement of blood in the arteries and capilaries occurring from dilatation of those vessels and from relaxation of its muscular walls. The calibre of blood vessels is regulated by the amount of nervous irritability, hence dilatation of its calibre is simply a withdrawal of its nerve force, or in other words vaso motor paralysis. The pulsations of the heart are increased in number, the result of interruption of the nervous influence of the pneumogastric nerve on that organ, causing increased rapidity of action. Coincidently with this, respiration is increased and more blood is thrown into the lungs. The arterial circulation increased, involves the venous plexus, and the lungs are taxed to their utmost to accommodate themselves to this condition. This rapid oxydation causes disturbance between respiration and circulation. Here the active cause is partial arrest in the innervation of the pneumogastric nerve, resulting in acceleration and retardation of the respiratory movements with engorgements of the pulmonary vessels; also diminishing the exhalations of carbonic acid, which amount to 25 per cent. in many cases. The hyperemic condition of the opthalmic artery is inferentially the same of the brain. This is confirmed by the intellectual aberration of the inebriate. When the medulla becomes affected, the organs of special sensation are involved and their functional activity is suspended. These are the prominent factors which point to paralysis as the essential lesion. Dr. Hays of Chicago, in a paper on this subject, remarks. "Whether we consider the dilatation of arteries, the increased supply of blood, the escape of the heart from its normal control, diminished volitional energy, disordered intellection, irrepressible emotional activity, diminished special sensibility, surpression of pulmonary exhalation and grandular secretion, loss of common sensation and motor power, separately or together, it will be apparent that each one points back to its ultimate factor, vaso motor paralysis, and diminished nerve power.

The doctor discussed the pathology of inebriety at length, show-

Refer to Dr. Hays' paper in Chicago Journal.

ing that it was a regular disease, having a cause, beginning, development, climax, also decline and extinction. Its treatment and cure as certain as that of other lessions of health, and concluding with the following summary: 1st. Inebriety is a disease of certain brain districts, and the nutritive functions which it controls. 2d. This disease may be excited, as produced by alcohol in variable quantities, depending upon some unknown condition of the body at the time of exposure.

3d. A weakened will power, with mental aberrations and tendency to inebriety not inherited, are manifestations of disturbance of the co-ordinating power of the nutritive functions. 4th. This disease is inherited, and exists as an alcoholic diathesis which may spring into activity, remain latent, or develop into other irregularities or functional diseases. 5th. Inebriety is the active cause of most of the nervous and functional diseases of the brain.

In the treatment of inebriety, Dr. Crothers remarked: It will be apparent to all from what we have said, that we have no specific treatment or remedy which will restore the integrity of the brain function. If the alcoholic diathesis is present, we may control it in a measure, by removing the exciting cause. If this diathesis is absent, we may prevent the increase of the disease by changing the habits and removing the active causes. The general treatment resolves itself into two divisions—reparative and restorative.

The reparative treatment lessens the intensity of the disease, enabling the patient to partially recover, and resume business again. The least hopeful classes are the periodic drinkers. They are subject to paroxysms similar to that of malarious poisoning; and during the frenzy seem abandoned by all restraint. After the attack, they make desperate and apparently successful efforts to save themselves but fall again as before. The asylums offer the only possibility for recovery or stay of this disease, by building and strengthening the moral and physical system against the recurring attacks This class comprises over forty per cent. of all the drunkards. It is asserted that there are over one hundred thousand inebriates in the United States who are periodic drinkers, and who are practically non-producers, growing worse every year. Under proper medical treatment at an asylum the extent and degree of these attacks may be so far lessened or broken up as to enable many of these men to regain their place in society and business. The doctor here detailed some illustrative cases of men who had been saved by treatment, and become valued citizens. He continued by saying that by the restorative treatment a patient regains his former health, and goes out into the world cured, in the same broad sense that a man having pleurisy or pneumonia becomes well again. He may be attacked again, but only from wanton exposure; all his power, strength and vigor have returned. All treatment must therefore resolve itself under these divisions. To accomplish either of these results you must have special surroundings and conveniences, such as can be found in but few private families. Hospitals and asylums, built for the special purpose of treating inebriates, remove the exciting cause, viz: alcoholism, which is of the first importance in the treatment of disease. 2d. They enable the patient to isolate himself from all excitement, and give opportunity for complete rest of the nervous system, giving nature better facilities to carry on the restorative process. 3d. They remove all care and responsibility from the patient's mind, except that of recovering his lost health, and make him a party with the physician to bring about this result. This in itself is a powerful factor in the treatment of any disease. 4th. Hospitals and asylums give the medical attendants complete control of the habits, care and surroundings of the patients. With these important and essential aids the treatment of inebriety is a practical reality, and is becoming a necessity in every community of the land. The victim of this disease has always a weakened physical system. The many functions and organs of the body have become poisoned, and impaired; the mental and moral forces, sympathizing with the diseased organism, lose control, and sink down to a level with the depraved and morbid appetites. Alcohol has paralyzed the functional organs, broken down the normal molecular nerve structure, perverted the healthy co-ordination of nutritive force, and in a greater or less degree, began a process of disorganization, ending in death unless checked. The treatment therefore must have refererence to removing the cause and building up the general system. Dr. Crothers described in detail the minutiæ of treatment at Binghamton and other asylums, which was substantially to improve and strengthen the physical and nervous system, and by raising the tone and vigor of the body and mind overcome the disease. The doctor remarked, the public treatment of the inebriate in both plan and spirit, is radically wrong. The cruelty to the insane of a century ago, is repeated in the poor outcast drunkard of to-day. All of our cities and larger villages have police courts, three-fourths of the business of which is to administer fines and punishments to poor diseased inebriates, who are broken down mentally and physically. The idea of true reformation never reaches this system; it is a prosecution of men who are not criminals, but sick patients needing medical and moral treatment. By this present effort to rid ourselves of this class, we substantially ruin them forever. The extent of this mistake is apparent in the statistics of the Albany penitentiary for the past twenty-three years, where 24,602 persons have been committed for crime; of this number 21,057 were inebriates. If these men had been treated in asylums, a large per cent. might have been cured, and become useful and honored citizens. But by this harsh treatment they were precipitated from the very doors of moral and physical health, and their ruin made more certain and fixed. The insane, blind and idiotic are cared for by asylums, but the outcast drunkard is held to strict accountability—a degree of ignorance which future generations will regard with amazement. The time is coming when we shall care for this class as we do the insane and idiotic. Then pauperism and crime will be lessened and our courts diminish. At the close of a noted trial in this city, an important order was issued containing this sentence,—"which inability does not appear to have arisen from causes beyond his control," etc. Had inebriety been understood, this sentence would never have been written, and the poor inebriate now to be tried for this impropriety, should not have been held responsible. Had his case been understood on the first manifestations of the disease, he would have been put under medical control, and his reputation and the cause of his client would not have suffered. * * * The number of inebriate asylums in the United States are eight, and the inmates number over sixteen hundred. This is but the advanced guard of the great army of five hundred thousand inebriates in this country who are passing forward towards the fountains of health, which science is opening slowly but surely. The results of treatment at these asylums are equal to that of the best insane asylums, varying from 40 to 50 per cent. of all the patients who are treated. At Binghamton over 40 per cent. have gone away and remained cured for three years, as long as any record has been kept of them. At other asylums the cures have been fully equal. The time of treatment averages from ninety to one hundred and twenty days. Many cases are incurable but all may be improved at the asylum.

Dr. Crothers concluded as follows: It is a source of much pride to know that our State is the first to recognize and take charge of inebriate asylums, of any Government in the world; also we have at Binghamton one of the largest and most complete asylums in existence. The problem of its success has been settled, and our State is foremost in this new field of progress. The conclusions from these facts are: 1st.—As medical men, in daily contact with this disease, we should seek to know its practical indications and general method of treatment. 2d—Alcoholism as a disease is increasing, and as a cause of organic lessons and diseases is spreading through all classes and societies. 3d—The public look to physicians for relief and instruction, and the problem of its cure and prevention must be worked out by them.

Dr. J. B. STONEHOUSE, Jr., made the following remarks on the subject:

I doubt the propriety of considering all drunkards as diseased; we should be careful in expressing our opinions in this matter Uncontrollable "thirst" may appear a symptom in three classes of dipsomania. The first is hereditary. The ancesters are found to be strongly nervous—insanity, paralysis, epilepsy or intemperance in the parent may predispose this disease. This is the *original* dipsomania of Bucknil and Tuke. The second variety is acquired, and is tracable directly to some lesion of the nervous centers, either from external injury, from previous disease or powerful mental impressions. The third variety is also acquired and is the least distinct of the three. It is produced by the excessive gratification of the desire for inebriants. It is in fact the continuation, the merging of this *habit* into a *diseased state* of mind, in which the moral tone and will are destroyed.

The first and second classes are probably accompanied by tissue changes, either congenital or acquired and more or less gross. The last class, however, is, as I have said, less definitely marked than the preceding, and it is the only class of drunkards in whose cases the question of responsibility becomes at all difficult to answer.

One word in regard to the Binghamton Asylum. I cannot agree with Dr. Crothers, in his unqualified praise of this institution and its managment. It has not been a success, and it is novel to hear a word spoken in its favor. I have yet to see the first patient who has been treated at Binghamton, and discharged permanently benefitted. I have however, known patients to speak bitterly of the time spent there as lost. It has been the common practice to send patients immediately after they become sober, to the city alone or with attendants, (and it amounts to about the same thing), to test their strength of will. Patient often object to this, pleading their inability to withstand the temptation, but in vain; they go, and the almost invariable result is another spree. A patient told me that he never failed to bribe the attendants, or to obtain money from outside by their aid. The patients sent to Binghamton, are generally of the wealthy class. To be sure, the State provides for a pauper class, but their admission is dependant upon the number of paying patients there, and therefore it is right to say that it is a private boarding house for gentlemen of means, who wish to recover from a debauch. Nine-tenths of the inmates can command at home the good air, magnificent scenery, unexceptionable food and medical attendance, which Dr. Crothers claims as the peculiar advantages of the institution. As an evidence of the lax managment here, I may say that only last year, a gentleman who had previously been under my care, committed suicide in Binghamton Asylum, from a self-administered dose of chloral.

These remarks are not intended in the least to apply to the administration of the last two or three months.

One other point: I have yet to find the authority, Gall and Spurzheim to the contrary notwithstanding, who claims for the dipsomaniac, a lesion confined to any settled cerebral region, such as may be the case in aphasia.

I would add to the formulæ which have been given, one which is said to be of service in dipsomania combined with dyspepsia—a combination of chloric ether and Tr. of nux vomica. But drugs will be found to be of little value as remedial agents.

Dr. E. H. Davis said that he had more faith in experience than in theories, in regard to the action of alcohol as a stimulant. It increases the activity, and gives tone to the heart, and in sickness it bridges over an interval, when without such stimulation the patient would die from exhaustion. This is the general experience of physicians. He could see no reason theoretically, in its chemical composition, why it should not act as a stimulant.

Dr. Crothers replied, that he believed it to be shown by late investigation that whatever force or stimulation is effected by alcohol when given therapeutically, is at the expense of vital strength.

Dr. Devol said that he had no confidence in brain regions. The Theories of Gall and Spurzheim have long been exploded, and were never founded on anatomical facts, as is seen in the locating important mental functions along the line of the great longitudinal fissure. He did not believe in calling the passion for alcoholics a a disease, and thought that their use could always be refrained from, not by running away from the temptation but by resisting it by an exercise of the will power.

The question of changing the time of meeting of the Society to afternoon, to accommodate members at a distance, was discussed, and finally laid on the table, it being the belief of some that the attendance would be diminished by the change.

